# A Low-Cost 3D Phenotype Measurement Method of Leafy Vegetables Using Video Recordings from Smartphones

**DOI:** 10.3390/s20216068

**Published:** 2020-10-25

**Authors:** Zishang Yang, Yuxing Han

**Affiliations:** 1 College of Electronic Engineering, South China Agricultural University, Guangzhou 510642, China; yzs@stu.scau.edu.cn; 2 Lingnan Guangdong Laboratory of Modern Agriculture, Guangzhou 510642, China

**Keywords:** plant phenotyping, machine vision, SfM, point cloud

## Abstract

Leafy vegetables are an essential source of the various nutrients that people need in their daily lives. The quantification of vegetable phenotypes and yield estimation are prerequisites for the selection of genetic varieties and for the improvement of planting methods. The traditional method is manual measurement, which is time-consuming and cumbersome. Therefore, there is a need for efficient and convenient in situ vegetable phenotype identification methods to provide data support for breeding research and for crop yield monitoring, thereby increasing vegetable yield. In this paper, a novel approach was developed for the in-situ determination of the three-dimensional (3D) phenotype of vegetables by recording video clips using smartphones. First, a smartphone was used to record the vegetable from different angles, and then the key frame containing the crop area in the video was obtained using an algorithm based on the vegetation index and scale-invariant feature transform algorithm (SIFT) matching. After obtaining the key frame, a dense point cloud of the vegetables was reconstructed using the Structure from Motion (SfM) method, and then the segmented point cloud and a point cloud skeleton were obtained using the clustering algorithm. Finally, the plant height, leaf number, leaf length, leaf angle, and other phenotypic parameters were obtained through the point cloud and point cloud skeleton. Comparing the obtained phenotypic parameters to the manual measurement results, the root-mean-square error (RMSE) of the plant height, leaf number, leaf length, and leaf angle were 1.82, 1.57, 2.43, and 4.7, respectively. The measurement accuracy of each indicators is greater than 80%. The results show that the proposed method provides a convenient, fast, and low-cost 3D phenotype measurement pipeline. Compared to other methods based on photogrammetry, this method does not need a labor-intensive image-capturing process and can reconstruct a high-quality point cloud model by directly recording videos of crops.

## 1. Introduction

Various types of leafy vegetables are an essential source of nutrients that people need in their daily lives. In order to increase the yield of plants, researchers use genetic breeding methods to find varieties that meet the quality requirements, of which quantification is a significant challenge. Traditional quantification mainly relies on manual measurement methods based on experience. With the development of agriculture, manual measurement methods have come to exhibit limitations in the rapid and accurate measurement of crops, which are time-consuming and cumbersome, and at the same time destroy crops. High-throughput phenotyping technologies provide the possibility to measure plant traits, and developing non-destructive and convenient measurement methods has become a significant challenge for researchers across various fields [1].

In the past ten years, researchers have used a wide variety of two-dimensional (2D) imaging technologies to obtain crop phenotypes. Such as on citrus measurement [2], durum wheat measurement [3], rape phenotypic parameters measurement [4], fruit detection [5], tree diameter measurement [6], and plant height assessment [7]. However, due to the lack of depth information in the 2D world, it is difficult to solve the occlusion problem, especially under field conditions, which is very common, making it difficult to obtain accurate structural information of the research object. In recent years, the use of three-dimensional (3D) sensing for plant phenotyping has increased [8]. Researchers in different fields use a variety of different 3D sensor-based technologies, which can be divided into two categories, namely, active and passive technologies [9]. Active sensors emit independent light sources, such as handheld laser scanning, structured light, ground laser scanning, and time of flight sensors [8]. Light Detection and Ranging (LiDAR) is one of the most widely used active sensors for phenotypic analysis. Wu et al. [10] used it to collect point cloud data of corn plants and achieved high-precision phenotypic parameter estimation. Moreno et al. [11] used on-board LiDAR to reconstruct vineyard crops and obtain vineyard biomass through data processing. In addition, Kinect is a universal and relatively cheap active sensor that can collect RGB-D data. Yang et al. [12] proposed a high-throughput seedling phenotypic analysis and processing method based on point cloud acquired by an RGB-D camera. Xiang et al. [13] used Kinect to collect sorghum point cloud data and obtained this plant’s phenotypic parameters through the skeletonization algorithm. Yang et al. [14] proposed a 3D shape measurement method for fruit tree canopies. Sun et al. [15] proposed a Kinect-based 3D phenotype calculation method for greenhouse plants. Wang et al. [16] evaluated the phenotypes of corn at multiple growth stages by comparing different 3D data collection methods. However, due to the low resolution of Kinect, it is difficult to accurately collect valid data in outdoor scenes.

The Structure from Motion (SfM) method, which is a passive method. This method collects images of the region of interest at different angles through the camera and then calculates the depth information of the target through the principle of triangulation. SfM can be used for the measurement of the phenotypic structure of plants, for yield estimation, and for good product prediction due to its simple use and its robustness. Sun et al. [17] reconstructed the 3D cotton boll map using the SfM method and obtained the number and location of cotton bolls through point cloud clustering and segmentation Xiao et al. [18] used the SfM method for the 3D reconstruction of sugar beets in different growth stages. Zermas et al. [19] used the SfM method to reconstruct the corn point cloud and obtained the phenotypic parameters by extracting the skeleton. Andújar et al. [20] used SfM technology to carry out 3D modeling of weed plants. Shi et al. [21] used the SfM algorithm to obtain a plant point cloud and used deep learning techniques to segment the parts of the plant. However, at present, SfM-related work often requires 30–50 images, the recording process is complicated and cumbersome, and the adjustment of the camera shutter and sensitivity parameters requires some experience. At the same time, the complexity of the field environment, such as the shelter of the parts of a plant, the influence of the wind on the steadiness of plants, and the lighting conditions, have brought challenges to this work.

Compared with previous work, this paper obtains crop data through mobile phone-based video recordings, making the most time-consuming data acquisition process handy in farmers’ pockets. The method does not require a complicated image shooting process, and can obtain crop phenotypic parameters through the proposed video processing pipeline. This included (1) using mobile phones to collect crop data via video recordings; (2) obtaining key frames containing crop areas in the video through an algorithm based on the vegetation index and scale-invariant feature transform algorithm (SIFT) matching; (3) reconstructing a single-crop point cloud using the SfM method; (4) obtaining the segmented point cloud and skeletonized model of leafy vegetables based on the clustering algorithm; (5) calculating the phenotypic characteristics of crops, such as the plant height, leaf area, and leaf length. Comparing the system measurement results to the manual measurement results, the RMSE values of the plant height, leaf number, leaf length, and leaf angle were 1.82, 1.57, 2.43, and 4.7, respectively; the plant height measurement with 94.8% accuracy, the leaf number estimation with 82.2% accuracy, the leaf length extraction with 83.4% accuracy, and the leaf angle extraction with 87.4% accuracy. The results show high correlations between manual measurements and the estimated values generated by the system proposed in this paper. In summary, this paper provides a convenient, fast, and low-cost 3D phenotype measurement process. After further development, this method can be applied to the analysis of other crops. 

## 2. Materials and Methods 

### 2.1. Experimental Design and Data Acquisition

**Figure 1:**
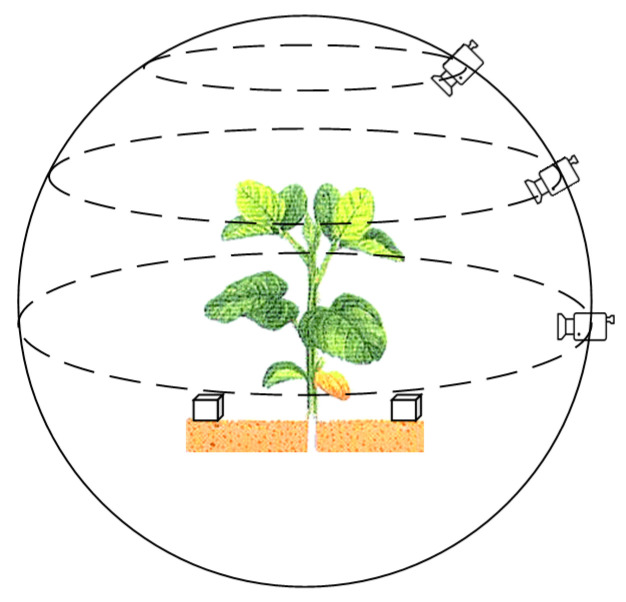
Video data collection method.

The experimental data was collected from a vegetable planting plot (35.28 N, 114.64 E) in Xinxiang, China. The time of collection was April 2020, and the crops leaf beet (*Beta vulgaris* L. var. *cicla* L.) were randomly selected for collection. Using a smartphone that has a camera with 20 million pixels, the video recording resolution was set to 2880 × 2160, the video frame rate to 30 fps, the exposure to automatic, and the focus mode to manually focusing before each shot. Recording around the crop was carried out in the most stable manner. In order to obtain as much plant information as possible, the recording distance was set to 30–45 cm of the center of the plant, and each plant was recorded three times at an angle of 0 degrees, 45 degrees, and 75 degrees relative to the ground plane. The recording method is shown in Figure 1.

After the video recording had been completed, the plant height, the leaf number, the leaf branch length, and the leaf angle were manually measured for each plant. Specifically, for the plant height, a rule was used to measure the distance from the above-ground part of the plant to the top of the plant canopy. For the number of leaves, the number was directly obtained by counting. For the leaf length, measuring tape was used to measure the length between the end of the main vein of the leaf and the stem. For the leaf angle, a protractor was used to measure the angle between the main vein of the leaf and the stem.

In order to obtain the dimensional relationship in space after reconstruction, during the recording process, a cube of a certain size (56 × 56 × 56 mm^3^) was placed on each side of the plant, as shown in Figure 1. By comparing the 3D reconstructed cubes, the relationship between the size of the plant model in the 3D virtual space and the real-world size was determined, so that the size of real-world plant could be obtained from the reconstruction of the plant point cloud.

### 2.2. Material Handling Pipeline

In this study, a low-cost 3D phenotype measurement pipeline for crops using video recordings taken with a smartphone was developed, which involved four steps:

(1) Obtaining key frames in the video that include plant areas through an algorithm based on the vegetation index and SIFT matching.

(2) Obtaining crop point clouds using the SfM method, and the pre-processing said point clouds.

(3) Using a clustering-based algorithm to obtain the segmented point cloud and the skeleton of point cloud.

(4) Obtaining the phenotypic traits of crops, including plant height, leaf surface area, and volume parameters.

#### 2.2.1. Acquisition of Key Frames

For the method of collecting data by recording videos, although it reduces the difficulty of data collection, the huge redundancy of the data poses a significant challenge to the reconstruction algorithm. For SfM-based point cloud generation algorithms, too many images will greatly increase the calculation time. Similarly, a small number of images or images of poor quality will result in missing or incorrect reconstructions. Xiao et al. [18] showed that for a single plant, 30–45 images could better balance the results between computation and quality. In this paper, the key frame is defined as the frame image in the video sequence that completely contains the crop area. The quality of key frames extracted from a video is one of the key factors affecting the quality of 3D crop reconstruction.

For videos recorded around crops using hand-held mobile phones, the video frames appear blurred due to shaking. Therefore, it is necessary to perform blur detection before extracting key frames. As we all know, there is less information in the boundary of a blurred picture compared to that of a clear picture. Therefore, calculation of the variance value of the second derivative of the picture can be used as the basis for judging whether the picture is blurred or not. The Laplacian operator has second-order derivability and can be used to calculate the border area in a picture, and the degree of blurriness of the image can be expressed as

(1) $$D\left(f\right)=\underset{y}{\sum }\underset{x}{\sum }\left|G\left(x,y\right)\right|$$

where G(x,y) is the convolution of the Laplacian operator at the pixel point (*x*, *y*) in image *f*.

After analyzing the captured video, the following two characteristics can be observed: (1) The foreground of the picture contains crops, and the background contains land. (2) The crops in the picture are almost still, and changes in the picture are mainly caused by the movement of the smartphone.

When randomly selecting a frame from the video, an obvious conclusion is that plants and land have different absorption characteristics for light in different wavebands, resulting in a clear distinction between the foreground and background colors.

The vegetation index mainly reflects the difference between the backgrounds of the vegetation and the soil in visible light, near-infrared band reflection, etc., and is used to enhance the difference between crops and the surrounding features [22]; therefore, it can effectively separate the backgrounds of green crops and the soil [23,24].

Common vegetation indices for visible spectra include the normalized vegetation index (NDI) [25], the excess green index (ExG) [26], the excess red index (ExR) [27], the excess green minus excess red index (ExGR) [28], and the vegetative index (VEG) [29]. For applying the normalization method of Formula (2) to an RGB color image, the calculation formula of each index is shown in Table 1.

(2) $$r=\frac{R}{R+G+B},\;g=\frac{G}{R+G+B},\;b=\frac{B}{R+G+B}$$

For the videos collected in this study, the foreground of the picture included green crops, and the background was similar to brown land. The research results of Guijarro et al. [30] show that using different vegetation indices can highlight specific colors and can obtain grayscale saliency maps with different features.

**Figure 2:**
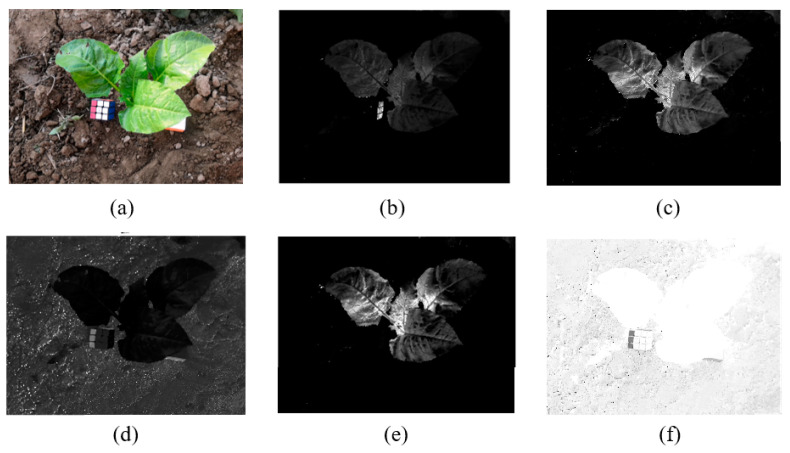
Images using different indices: (**a**) original image; (**b**) NDI image; (**c**) ExG image; (**d**) ExR image; (**e**) ExGR image; (**f**) VEG image.

In this paper, the images under various vegetation indices were verified, showing that ExGR has better results for the vegetable images collected in this study (Figure 2).

**Figure 3:**
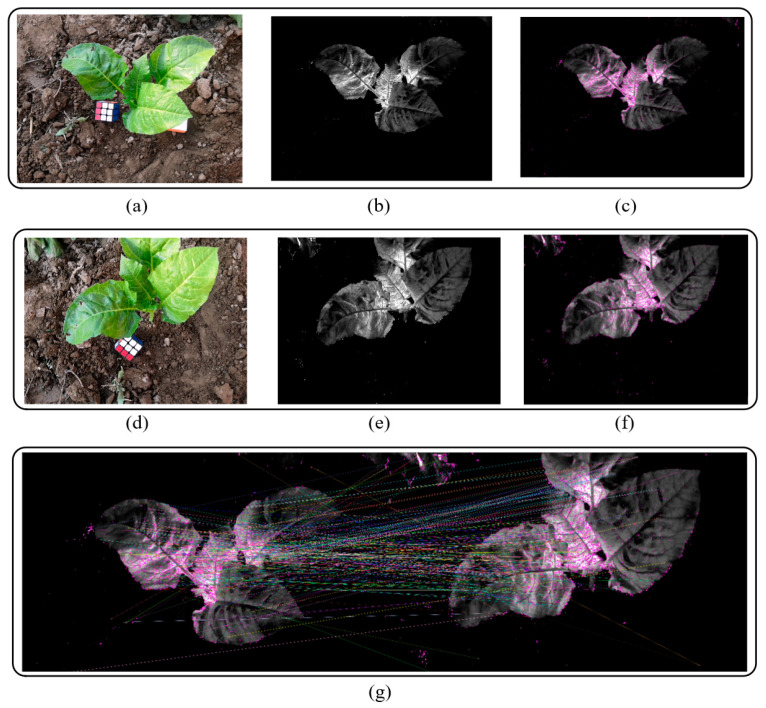
Key frame extraction. (**a**,**d**) original images; (**b**,**e**) ExGR images, (**c**,**f**) scale-invariant feature transform algorithm (SIFT) feature images; (**g**) matching image.

The SIFT [31] is one of the most robust and widely used algorithms in image local feature extraction algorithms. SIFT features have scale and rotation invariant characteristics, with excellent performance in complex changing environments such as image translation, scaling, and rotation, as well as certain robustness to lighting and noise. It is often used in image stitching, point cloud model reconstruction, and other algorithms. For this study, the purpose of generating key frames was to use the SfM algorithm reconstruction in the subsequent steps. Therefore, a method based on vegetation index and the SIFT matching algorithm was proposed to obtain the key frames of videos. The basic pipeline includes the following steps:

(1) Let I be a frame image in the video. For a video with n frames of image $S=\left\{I_{i},\;i=1,2,3\ldots n\right\}$, suppose the expected number of key frames is m. Then, divide the video S into m sets at equal intervals from the first frame. Taking the first frame of each set as the starting frame, first determine whether the current frame is blurred, and if so, directly remove the current frame and then determine the next frame, until the video frame in this set has been judged. The frames left in all sets will then be clear frames only.

(2) Apply the ExGR method to the video frames in each set to obtain the grayscale crop saliency map $I^{\prime }$.

(3) Calculate the SIFT features of each crop saliency map in all sets. Taking the first saliency map $I_{1}^{\prime }$ of the first set as the first key frame, starting from the second set, match all of the saliency maps in the current set with the feature points of the previous key frame. Use the frame with the most matching points in the current set as the new key frame. Repeat the current step until *m* sets are calculated and *m* key frames are obtained. Figure 3 shows the results of image graying and SIFT matching. The calculation formula is

(3) $$I_{key}^{i}=max\left\{Num_{SIFT}\left(I_{l}^{\prime },I_{key}^{i-1}\right),\;I_{l}^{\prime }\in \;{\left\{I^{\prime }\right\}}_{i},i=2,3\ldots m\right\}$$

(4) Use the corresponding frames of the *m* key frames in the original video as the reconstructed image sequence to reconstruct the 3D point cloud model of the crops.

#### 2.2.2. Generation and Preprocessing of a Point Cloud

**Figure 4:**
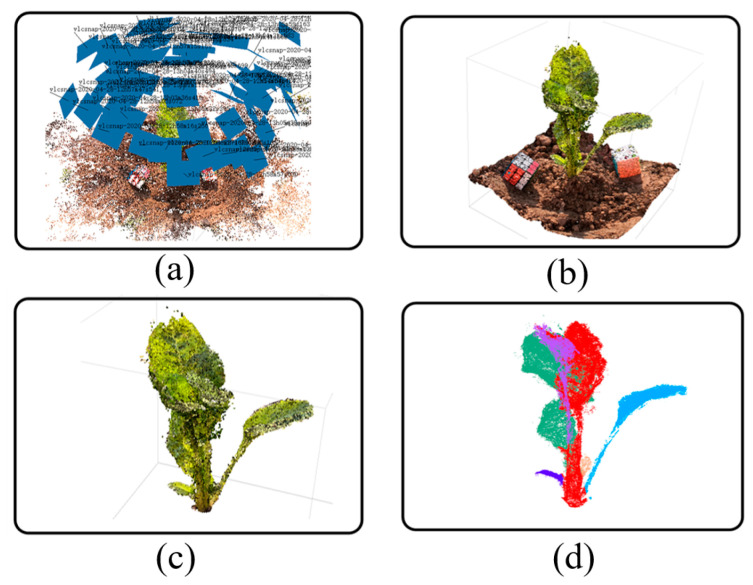
The process of generating a point cloud. (**a**) SfM reconstruction, (**b**) dense point cloud, (**c**) point cloud after ground removal and filtering, (**d**) point cloud segmentation.

For the obtained image sequence, the SfM algorithm is used to obtain the sparse point cloud, and on this basis, the Multi View System (MVS) algorithm is used to reconstruct the sparse point cloud into a dense point cloud. The point cloud generation process in this paper is outlined in Figure 4, showing the results of the SfM reconstruction for the image sequence extracted from the video (Figure 4a), the results of the dense point cloud generation (Figure 4b), the results of the ground removal and the filtered point cloud (Figure 4c), and the results of the point cloud segmentation (Figure 4d).

Popular tools include openMVG, VisualSfM, and Cloud Compare [9]. In this study, Agisoft Metashape (version 1.6, Agisoft LLC, St. Petersburg, Russia) software was used to calculate the SfM and MVS values and to generate the point cloud model. For the point cloud generation pipeline, the software includes two steps, namely, image alignment and building a dense point cloud. In this study, for image alignment, the calculation accuracy was set to a high level, and the upper limit of the feature points and matching points of the image were set to 40,000 and 10,000, respectively. For building dense point clouds, the point cloud quality was also set to a high level, while applying a slight depth filter to reduce the possibility of erroneously eliminating important features.

A reconstructed point cloud contains not only plants, but also the ground. Due to the way of recording around the plant, the reconstructed point cloud model can, in most cases, be approximately perpendicular to the horizontal plane of the 3D space, which avoids the rotation and translation of the point cloud in the 3D space. However, the ground is redundant for subsequent algorithm steps. Therefore, the ground needs to be detected first. In this paper, the random sample consensus (RANSAC) algorithm was used to fit the ground plane [13,18]. After detecting the ground, the direction perpendicular to the ground was defined as the Z-axis, and the point cloud corresponding to the ground was deleted. However, because the real ground is not flat, deletion is typically inaccurate and often requires manual involvement. Specifically, using MeshLab software [32] deletes redundant point clouds and adjusts the direction of the calculated normal vector through mouse interaction. Finally, the content above the ground was reserved as the region of interest for the subsequent calculation of plant phenotypes.

Due to the high resolution of the captured images, reconstructed high-precision point clouds often contain hundreds of thousands, or even millions of orders of magnitude, which results in a huge amount of calculations for point cloud processing [33]. In order to improve the efficiency of point cloud processing, MeshLab software is commonly used to simplify the point cloud, reducing the number of point clouds per plant to approximately 10,000–30,000, which effectively reduces the amount of calculations and has little effect on the accuracy of point cloud processing [10].

At the same time, due to the influence of the outdoor environment, the generated point cloud model will contain some discretely distributed noise. If the noise is not processed, it will affect the subsequent calculations. Therefore, in this study, the radius-based outlier filter was adopted [33], and the neighborhood value was manually set to perform noise point filtering. Finally, a smoother point cloud model was obtained.

#### 2.2.3. Point Cloud Skeleton Extraction and Segmentation

The point cloud model not only has a large number of data and high amount of redundancy, but it is also difficult to directly obtain effective plant phenotypic parameters [34]. The skeleton can not only reflect the topological information of a shape, but can also describe the geometric information of said shape [35]. Therefore, in this study, the point cloud model was segmented and skeletonized, and the phenotypic information was obtained from them.

A point cloud skeletonization and segmentation algorithm based on slice clustering was used in this study [13,19]. First, the point cloud was divided into slices from the Z-axis direction with a certain height ∆h. For each slice, a clustering algorithm was used to find possible centroids, and then the centroid was used as the node of the point cloud skeleton. At the same time, the connected Euclidean clusters [36] were linked between adjacent layers to ensure connectivity, thereby obtaining a rough branch of the point cloud skeleton. For the obtained branches, due to clustering not being an accurate calculation process, it may contain false branches, and so a branch pruning algorithm based on a number threshold was further adopted to delete branches with less than a specified number of nodes. Finally, cubic B-spline fitting was performed on the skeleton to obtain a continuous and smooth skeleton.

In the process of calculating the centroid in the skeleton generation step, the correspondence between the centroid point and the original point cloud was recorded, so that the correspondence between the skeleton branch and the original point cloud could be obtained and then segmented according to different branches of the plant. On the basis of obtaining the skeleton, the skeleton was up-sampled, and more skeleton nodes were obtained. Then, each node was taken as a seed point, and the clustering method was used to obtain the adjacent point cloud [19]. Finally, the point clouds corresponding to the nodes were combined, so that different branches of the plant could be segmented.

### 2.3. Verification and Statistical Analysis

#### 2.3.1. Size Transformation of the Point Cloud

In order to obtain the relationship between the plant point cloud in the 3D virtual space and the plant in the real world, during the recording process, cubes of a certain size were placed on each plant. To ensure the accuracy, two cubes were used to avoid loss during the reconstruction of the point cloud. By measuring the size of the cube in the 3D virtual space, the scaling factor $k=L_{real}/L_{virtual}$ could be obtained, where $L_{real}$ is the value of the cube in the real world and $L_{virtual}$ is the value of the cube in the point cloud space. Therefore, through the scale factor, the real-world measurement results could be easily obtained from the virtual world.

#### 2.3.2. Calculation of the Phenotypic Parameters

In this paper, the following phenotypic traits were obtained: the plant height, the leaf number, the leaf branch length, the leaf angle, and the leaf surface area.

Plant height refers to the total height of the above-ground part of the plant, while the number of leaves is often used to characterize the amount of vegetable biomass. The leaf branch length is defined as the length of each individual leaf branch from the bottom to the end of the blade, while the leaf angle is defined as the angle between the middle stem of the leaf and the ground. Finally, the leaf surface area characterizes the size of the leaf. The calculation method of each parameter is as follows:

For the plant height, when the plant is nearly perpendicular to the ground, it is the Euclidean distance from the ground to the top of the plant. In this case, it can be obtained by subtracting the minimum value from the maximum value in the Z-axis direction of the point cloud model. The plant height $H=\left|z_{max}-z_{min}\right|$, where $z_{max}$ and $z_{min}$ are the maximum and minimum values of the *Z*-axis coordinates in the point cloud. When there is a certain angle θ between the plant and the ground, the plant height $H=\left|z_{max}-z_{min}\right|/sin\;\theta$.

For the leafy vegetables collected in this experiment, there was no obvious boundary between the leaves and the stems, so the number of skeleton branches obtained could be used to represent the number of leaves. Therefore, the leaf number can be calculated by the number of skeleton branches.

The leaf length can be calculated by using the length of the skeleton branch. For a branch that includes *n* nodes $v_{i}$(*i* = 1…*n*), the branch length is the sum of the distances between the nodes. For each leaf length $L_{b}$,

(4) $$L_{b}=\overset{n-1}{\underset{i=1}{\sum }}\left|\vec{v_{i}v_{i+1}}\right|.$$

For the leaf angle, the ground normal vector α if first calculated, followed by the tangent vector γ of the skeleton branch; the leaf angle θ is

(5) $$\theta =arccos\left(\frac{\alpha \gamma }{\left|\alpha \right|\left|\gamma \right|}\right).$$

#### 2.3.3. Error Measurement

The calculated phenotypic parameters were compared to the manual measurement results to prove the accuracy of the system. The root-mean-square error (RMSE) and the mean absolute percentage error (MAPE) of the measurement results can be calculated as follows:

(6) $$RMSE=\sqrt{\frac{1}{n}\overset{n}{\underset{i=1}{\sum }}{\left(x_{si}-x_{mi}\right)}^{2}}$$

(7) $$MAPE=\frac{1}{n}\overset{n}{\underset{i=1}{\sum }}\frac{\left|x_{si}-x_{mi}\right|}{x_{mi}}\times 100\%$$

where $x_{si}$ is the system measurement result and $x_{mi}$ is the manual measurement result.

## 3. Results

In order to verify the effect of the method proposed in this paper, 15 green leafy vegetable plants with different shapes were randomly selected to record the videos, and then the phenotypic parameters were obtained through a series of processing pipelines. The computer hardware and software parameters used in the experiment were an Intel i7-8750H @ 2.20GHz CPU (Intel Corp., Santa Clara, CA, USA), a 16 GB memory, a GTX1060 GPU, a Window 10 v. 2004 operating system, Python programming language, and libraries such as OpenCVand VTK (Kitware Inc., NY, USA) for algorithm development.

### 3.1. The Key Frame Extraction Results

**Figure 5:**
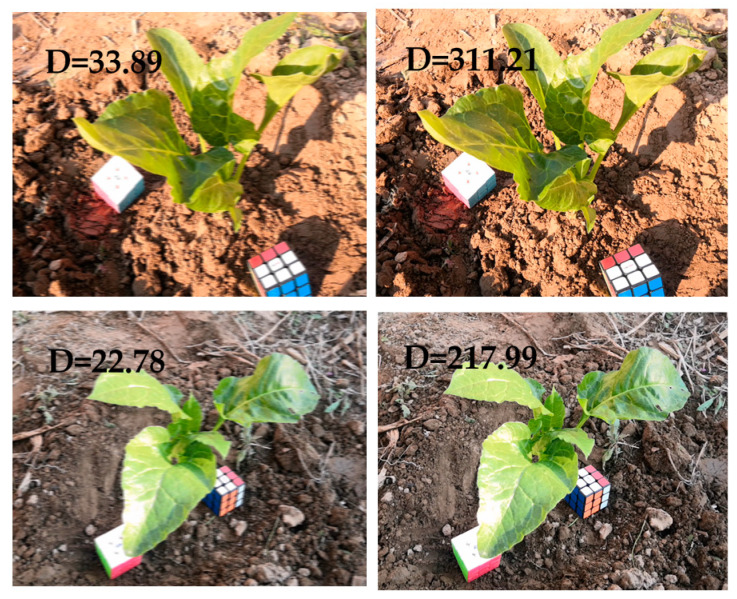
Comparison of blurred images (left) to normal images (right). D represents the degree of blurriness of the image.

For extracting the key frames, the key steps that affect the results are the image blur detection and the SIFT matching process. For this experiment, the reason for the blur of the video frame was the jitter during the recording process, and the actual degree of jitter was affected by the recording level and equipment. Therefore, the threshold value of the degree of blurriness was related to the object photographed each time. For each recording session, the average blur value was calculated from 10 images without visible blur. The threshold was set to three-quarters of the average blur value. After comparison, there was no obvious blur for images larger than this threshold. Figure 5 shows the comparison between blurred and normal images.

After removing the blurry image frames, the vegetation index was calculated for each frame image to obtain a grayscale crop salient image. On this basis, the SIFT features of each salient image were calculated, and the feature points were matched in sequence according to the designed steps.

### 3.2. Point Cloud Processing Results

**Figure 6:**
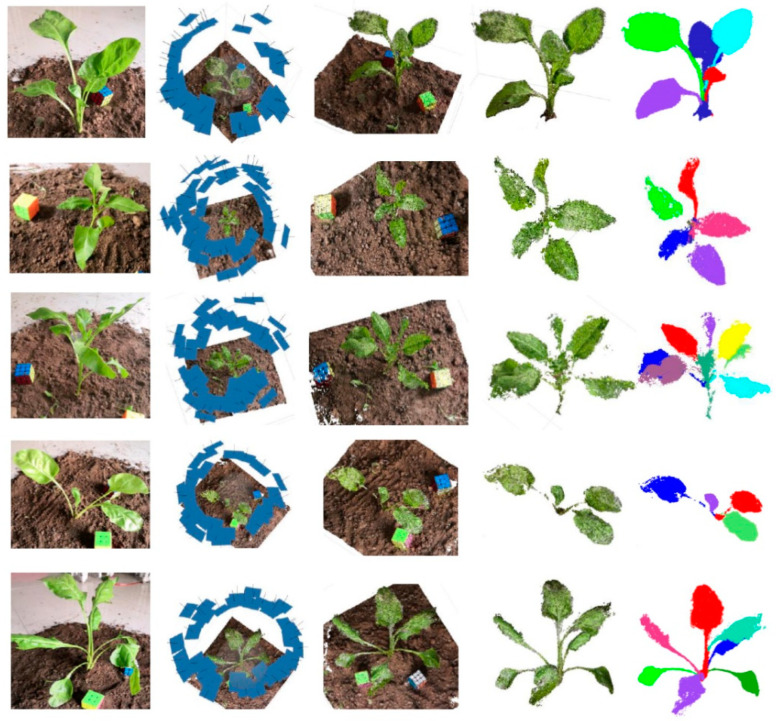
The results of the point cloud processing.

On the basis of obtaining the plant image sequence, reconstruction software was used to generate the point cloud, and then the pipeline proposed in this paper was used to obtain the point cloud skeleton and the segmented point cloud. The results show the excellent performance of the key frame extraction method proposed in this paper. Figure 6 shows the five sets of results of this experiment, including, from left to right, the original images of the crop, the 3D position results of the image sequence extracted from the video, the results of the dense point cloud, the results of the ground removal and the filtered point cloud, and the results of point cloud segmentation.

### 3.3. Phenotypic Parameter Measurement Results

For the obtained point cloud model, the plant height, the number of leaves, the length of the leaf branches, and the angle of the leaves were calculated according to the designed pipeline. In order to verify the accuracy of the designed algorithm, the values obtained by the system were compared to the results of the manual measurements. Finally, a regression analysis was performed on both sets of data, and the root-mean-square error and the mean absolute percentage error were calculated.

**Figure 7:**
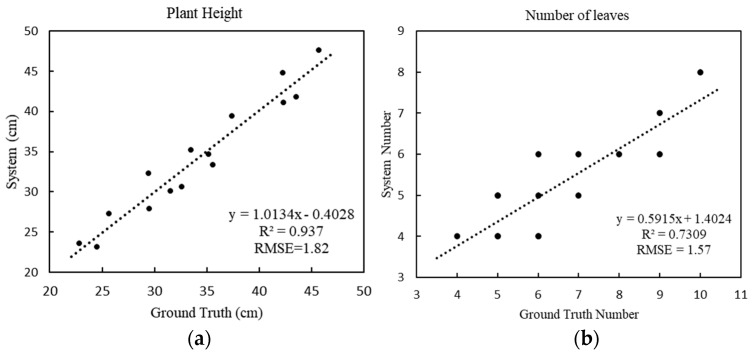
Regression analyses comparing actual height versus estimated height (**a**), and actual leaf number versus estimated leaf number (**b**).

For plant height (Figure 7a), the RMSE is 1.82 cm and the MAPE is 5.12%, and the fitting equation shows that the system has a strong explicit correlation between the estimated height and the actual height, which shows the accuracy of the system’s calculation of plant height. For the number of leaves (Figure 7b), the RMSE is 1.57 and the MAPE is 17.78%, which means that the system results deviate from the actual results. The results of the system were estimated by calculating the number of skeleton branches of the plant; a single branch of some green leafy vegetables grew multiple leaves, causing inaccuracy of the skeleton branch calculations, thus resulting in the system being biased.

**Figure 8:**
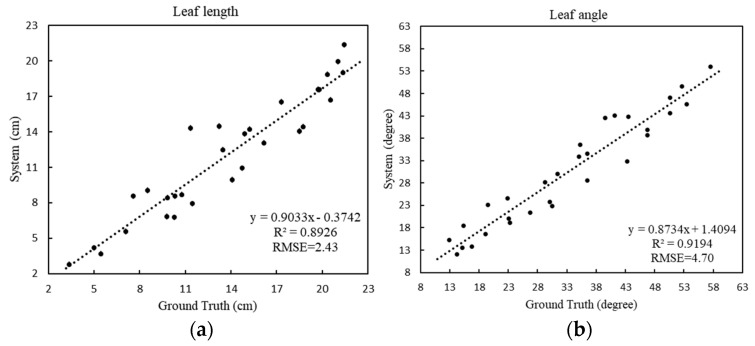
Regression analyses comparing actual leaf length versus estimated leaf length (**a**), and actual leaf angle versus estimated leaf angle (**b**).

For the leaf length (Figure 8a), the RMSE is 2.43 cm and the MAPE is 16.61%, showing that the proposed method can provide accurate blade length estimations. For the leaf angle (Figure 8b), the RMSE is 4.70 degrees and the MAPE is 12.61%, which indicates the accuracy of the skeleton extracted by the model, showing that the obtained skeleton can effectively characterize the spatial structure of a plant, and that the method can handle leafy vegetable crops of various structures.

## 4. Discussion

In this paper, a novel method for acquiring in situ vegetable 3D phenotypes by using smartphones to capture videos was proposed. The method includes the extraction of key frames from videos, the generation and processing of point clouds, and the calculation of phenotypic parameters. The experimental results show the accuracy of the proposed method for measuring the 3D phenotypes of leafy vegetables. Compared to other technologies such as LiDAR and laser scanning [37,38], this method has a lower cost and has more reliable portability. However, it should be noted that there are still certain limitations to the proposed method.

For the process of collecting video data through smartphones, the jitter caused by smartphone movement makes the extracted images appear fuzzy, so a fuzzy evaluation method was designed. By judging and removing the blurred images, the accuracy of subsequent point cloud generation is guaranteed. According to the outcome of this experiment, recording videos through an external stable device or a smartphone with a stronger anti-shake function can effectively reduce the jitter problem caused by human factors. In addition, the influence of light and wind also brings challenges to the generation of point clouds. For the former, too much or too little light can reduce the quality of the captured video, resulting in the inability to obtain a sufficient number of feature points [33]. In the latter case, the wind causes vibrations of the plant, which results in failure of the feature point matching, and further leads to the problem of missing point cloud generation [39]. Therefore, under certain external conditions, it is necessary to improve the external lighting conditions and to use wind-shielding devices to reduce the impact of external conditions.

For key frame extraction, normally, the detection of the degree of blurriness of an image can remove most of the blurred image. However, for different plants, the threshold of blurriness needs to be adjusted according to different videos. At the same time, a key frame detection method was proposed in the paper based on ExGR and SIFT matching. For ExGR, the experimental results show that this method can effectively separate green leafy vegetables and the land background, and at the same time can be robust in terms of a variety of green leafy plants [30,40]. The SIFT matching method adopted in this paper can ensure that the reconstruction results of the obtained image frame are relatively optimal. At the same time, the steps of ExGR calculation can effectively reduce the interference of the background to SIFT matching, and can ensure that the obtained key frames contain crop areas.

For the generated point cloud, thanks to the rotation of the recording around the plant, the reconstructed plant point cloud model can be approximately perpendicular to the horizontal plane of the 3D space in most cases. However, due to the growth factors of the plant itself, there may be an angle between the point cloud after reconstruction and the horizontal plane of the 3D space. Therefore, a more effective method is to use the Principal Components Analysis (PCA) algorithm to calculate the main direction vector of the plant after removing the ground [13], and then to use the main direction vector to rotate and translate the point cloud model in the 3D space, thereby increasing the accuracy of the calculation of the phenotypic parameters.

As we all know, accurate segmentation of point cloud models is still a huge challenge. In this paper, the skeleton of the point cloud was obtained by clustering point cloud slices in the first instance, and then the segmented point cloud model was obtained by sampling and clustering the skeleton. However, for randomly selected vegetable materials, the performance of the algorithm shows huge variation. For plants with overlapping leaves, the clustering algorithm leads to the loss of skeleton nodes, resulting in the inability of branches to become effectively connected. Furthermore, individual leaves cannot be separated from the point cloud, which affects the accuracy of phenotypic parameter calculation. In recent years, some researchers have studied the skeleton extraction of plant point cloud models [10,13,19]. Similarly, skeleton extraction and segmentation through mesh models provides different solutions [41]. Exploring the effective segmentation of point clouds is still a topic that needs to be further studied.

For the method of obtaining the scaling factor k in this paper, the measurement results of the cube in the 3D space mainly affects the accuracy of the value. In order to ensure the accuracy of the value, this experiment placed two cubes at different positions to avoid measurement errors caused by incomplete 3D cube reconstruction. At the same time, to avoid errors caused by manual measurement, multiple measurements were taken on two cubes, and use the average value as the official measurement result. Furthermore, to avoid the influence of artificial factors on the results, planes fitting can be applied to the 3D point cloud cube model, and the distance between two parallel planes can be calculated as the measurement result.

For the experiment, the video duration of each crop is within 2 min. The 3D point cloud reconstruction part occupies most of the pipeline time and requires a large amount of computing resources. For the hardware used in this experiment, the entire process takes 90 to 150 min without a GPU, and it takes about 30 to 55 min with a GPU. The factors that affect the time consumption include 3D reconstruction parameters and the number of reconstructed point clouds. In order to reduce time consumption, a lower reconstruction quality and more radical point cloud simplification parameters can be adopted. With the development of computer vision technology, it is expected that a more effective SfM algorithm will be established in the future to reduce time consumption.

## 5. Conclusions

In order to achieve low-cost 3D crop phenotype measurement, this paper proposed a processing pipeline based on the SfM method for processing the video recordings of smartphones. This method does not require recording multiple images from different angles and can reconstruct a high-quality point cloud model by only recording videos around the crops, which avoids the complicated data collection process. First of all, a video of the crop is recorded using a smartphone, and then technology for obtaining the key frame of the plant area from said video is studied. On this basis, the plant point cloud is obtained through the SfM reconstruction of key frames. In order to obtain plant phenotypic parameters, it is proposed to process the plant point cloud through two steps, namely, point cloud skeleton extraction and point cloud segmentation. Finally, the phenotypic parameters such as plant height, leaf number, leaf branch length, and leaf angle are obtained from the point cloud and skeleton. Comparing the system measurement results to the manual measurement results, the RMSE values of the plant height, leaf number, leaf length, and leaf angle were 1.82, 1.57, 2.43, and 4.7, respectively; the plant height measurement with 94.8% accuracy, the leaf number estimation with 82.2% accuracy, the leaf length extraction with 83.4% accuracy, the leaf angle extraction with 87.4% accuracy. Therefore, it can be concluded that the system has a high measurement accuracy. The acquired phenotypic parameters can provide effective guidance for crop breeding and growth management. Moreover, this method can provide 3D reconstruction and parameter extraction during the entire growth period of leafy vegetables and can also be applied to other crops.

## Figures and Tables

**Table 1:** Common vegetation indices.

	Type	Definition
1	NDI	(g−r)/(g+r)
2	ExG	2g−r−b
3	ExR	1.48r−g
4	ExGR	ExG−ExR
5	VEG	$g/\left(r^{0.667}b^{0.333}\right)$

NDI, normalized vegetation index; ExG, excess green index; ExR, excess red index; ExGR, excess green minus red index; VEG, vegetative index.
